# Iodine and Thyroid Maternal and Fetal Metabolism during Pregnancy

**DOI:** 10.3390/metabo13050633

**Published:** 2023-05-06

**Authors:** Charles Mégier, Grégoire Dumery, Dominique Luton

**Affiliations:** Assistance Publique-Hôpitaux de Paris, Service de Gynécologie-Obstétrique, Hôpital Bicêtre, Université Paris Saclay, 94270 Le Kremlin-Bicetre, France

**Keywords:** iodine, thyroid, pregnancy, placenta, fetus, hypothyroidism, hyperthyroidism

## Abstract

Thyroid hormones and iodine are required to increase basal metabolic rate and to regulate protein synthesis, long bone growth and neuronal maturation. They are also essential for protein, fat and carbohydrate metabolism regulation. Imbalances in thyroid and iodine metabolism can negatively affect these vital functions. Pregnant women are at risk of hypo or hyperthyroidism, in relation to or regardless of their medical history, with potential dramatic outcomes. Fetal development highly relies on thyroid and iodine metabolism and can be compromised if they malfunction. As the interface between the fetus and the mother, the placenta plays a crucial role in thyroid and iodine metabolism during pregnancy. This narrative review aims to provide an update on current knowledge of thyroid and iodine metabolism in normal and pathological pregnancies. After a brief description of general thyroid and iodine metabolism, their main modifications during normal pregnancies and the placental molecular actors are described. We then discuss the most frequent pathologies to illustrate the upmost importance of iodine and thyroid for both the mother and the fetus.

## 1. Introduction

Iodine is an essential mineral nutrient, mainly involved in thyroid metabolism. It is a key component of thyroid hormones (TH), which are essential for kidney, liver and brain metabolism. A healthy adult individual contains 15–20 mg of iodine, 70–80% of which is located in the thyroid gland [1,2]. Iodine deficiency (ID) is frequent worldwide, affecting approximately 40% of the world’s population [1]. Pregnancy tends to worsen ID, and around two third of European pregnant women are iodine deficient [3]. Maternal thyroid metabolism can be heavily impaired during pregnancy because of ID, auto-immune disease or pregnancy-related changes. As TH are mandatory for fetal development, the placenta plays a crucial role during the pregnancy [2,4]. Indeed, it provides the fetus with maternal TH as long as the fetal thyroid is not fully functional [5]. It also supplies the fetal thyroid with iodine in order to synthesize fetal TH from the end of the first trimester [5,6]. Here, we provide a narrative review of the data concerning normal and pathological iodine and thyroid metabolism during pregnancy.

## 2. General Iodine and Thyroid Metabolism

### 2.1. Iodine Absorption and Excretion

Iodine’s primary source is dietary intake. Animals and plants of marine origin usually present the highest amount of iodine among types of food because they concentrate iodine from seawater. Unfortunately, dietary iodine intake is rather low all around the world. Indeed, most of the consumed sources only provide 3–80 µg per serving, whereas WHO recommends a daily dose of 150 µg of iodine [7,8]. The main iodine sources in many western countries are bread and dairy products, and the low iodine amount of those aliments explains the high prevalence of ID in those areas [9]. The level of iodine in bread is linked to its addition in flour, while that of dairy products comes from the products themselves. In Asian countries—especially in Japan—consumption of seawater food diminishes the prevalence of ID [10]. In order to maximize dietary iodine intake, cooking salt is iodized worldwide [11,12]. This constitutes a major source of additional iodine [13,14]. However, its contribution alone is not completely sufficient to ensure adequate intake [15].

In addition to dietary intake, iodine is efficiently recycled by the human body. Bloodstream iodine that is not imported into the thyroid or that comes from TH degradation in peripheral tissues is accumulated in the salivary glands and stomach [16]. Both can excrete iodine, increasing the pool that will be absorbed by the duodenum, jejunum, and ileum. 

One of the main proteins involved in iodine metabolism is the NIS symporter (Sodium/Iodine Symporter, SLC515), a transmembrane glycoprotein weighting 90–100 kDa [17]. NIS is one of the most effective iodine transporters as it has a twice-higher affinity for iodine than the other ones. With its 13 transmembrane domains, it can transport two sodium cations (Na^+^) for each iodine anion (also known as iodide, I^−^). The transport direction depends on the sodium gradient, which is created by Na^+^/K^+^ ATPase. The energy obtained by exporting sodium—which follows the electrochemical gradient of Na^+^/K^+^ ATPase—is used to import iodine against its own electrochemical gradient. 

In the salivary glands, NIS is located on the ductal cells’ basal membrane. It imports iodine from the bloodstream into the salivary gland cells. Iodine can then diffuse by osmosis to the apical surface, where it is taken in charge by other transporters such as CFTR, Anoctamin 1 (Tmem16A) and/or Pendrin (SLC26A4) in order to release it into the oral cavity thus increasing the diet pool [18,19]. The intracellular iodine pool can influence its own transport. For instance, Pendrin can be activated by a high concentration of intracellular iodine [20]. A similar phenomenon takes place in the stomach, where the accumulation of NIS on the basal membrane of mucin-secreting and parietal cells suggests iodine transfer from the bloodstream to the stomach cells [21]. The export from those cells to the gastric fluid is realized by CFTR and Anoctamin 1 [22]. 

Dietary and recycled iodine accumulates in the gut lumen. They are mainly absorbed in the duodenum, jejunum, and ileum [23]. NIS once again plays a major role in iodine import, this time located on the apical side of enterocytes, allowing import into the cells in a manner similar to the one in the salivary glands (working in conjunction with the Na/K ATPases). How iodine is exported from the gut cells to the bloodstream remains unclear, but chloride and iodine symporters located on the basal surface of enterocytes, such as Chloride Channel 2 (ClC-2), may have a role in this process [16,24].

When dietary iodine intake is adequate, more than 80% of iodine is passively excreted in the urine via the glomerulus [25]. When it is inadequate, the kidney is the ultimate location to reabsorb iodin. This reabsorption probably involves the usual transporters (NIS, CFTR, Pendrin, Anoctamin 1), but the iodine metabolism in the kidney has not been fully elucidated [26,27,28,29].

Urinary iodine concentration results in a balance between dietary intake, thyroidal metabolism, and renal function. It is the most convenient method to assess the iodine status. The best urinary iodine excretion evaluation method applied to a population consists of a 24 h urinary collection [30,31]. Unfortunately, it has low individual value. A population with correct intakes should present a urinary iodine concentration between 100–199 µg/L [32]. ID is suspected when UIC is <100 µg/L in non-pregnant women (severe if IUC <20 µg/L, moderate if IUC = 20 − 49 µg/L and mild if IUC = 50 − 99 µg/L). The risk of iodine-induced hyperthyroidism exists if the IUC is >300 µg/L [33]. 

### 2.2. Thyroid Metabolism

Iodine (I) is the oxidized form of iodide (I^−^) and the main component of TH. Those TH are phenolic rings bound with an ether link. They can be iodinated in three positions, forming 3,5,3′-tri-iodo-L-thyronine (also known as triiodothyronine, T3) or four positions, forming 3,5,3′,5′-tetra-iodo-L-thyronine (also known as thyroxine, T4) [17,34]. 

I^−^ is imported from the blood circulation into the thyrocytes (thyroid follicular epithelial cells) by NIS–located on their basolateral side–and released into the follicular lumen by Pendrin, and eventually Chloride Channel 5 (ClC5) and/or Anoctamin. The fraction of iodine imported into the thyroid varies according to the iodine intake [2]. Less than 20% of the absorbed iodine is taken up by the thyroid when the dietary intake is adequate, whereas more than 80% can be imported in cases of chronic ID.

Into the follicular lumen, accumulated I^−^ is oxidized into diatomic iodine (I2) by Thyroid peroxidase (also called thyroperoxidase or iodide peroxidase, TPO) [17,34]. TPO is located on the apical side of the thyroid epithelial cell and faces the follicular lumen. The energy needed for this oxidation is provided by hydrogen peroxide (H_2_O_2_), generated mainly by a dual oxidase 2 and additionally by a dual oxidase 1 [35,36]. Dual oxidase 1 and 2 both belong to the NADPH oxidase family.

Iodine is afterward covalently linked on three or four positions of tyrosyl residues on Thyroglobulin (TG) [34]. TG is synthetized in the thyrocytes by the rough endoplasmic reticulum ribosomes and exported by exocytosis into the follicular lumen [34]. Its concentration in the follicular lumen is approximately 100–400 mg/mL. Iodine binding on TG creates iodinated TH intermediates, 3-mono-iodotyrosine (MIT) and 3,5-di-iodotyrosine (DIT). In order to form T3 and T4, MIT and DIT are respectively coupled on the TG polypeptide backbone by TPO. Follicular TG is re-internalized into thyrocytes by endocytosis and proteolyzed. This frees thyroid hormones from the polypeptide backbone. T3 and T4 are then secreted into the blood stream by transporters such as (Mono-Carboxylate Transporter 8 (MCT8) at the basolateral membrane of thyrocytes [34]. 

T4 is considered to be a prohormone, as it is three to four times less potent than T3 and can be converted into T3 by deiodinases [37]. The ratio of T4 to T3 released into the bloodstream is approximately 14:1, and the major part of T3 in the human body is thus generated in peripheral tissues by the deiodination of T4.

In the blood stream, TH are in the vast majority (more than 99%) transported by proteins such as albumin, Thyroxine-Binding Globulin (TBG) or transthyretin [38]. Only a fraction of TH are unbound; they are called free T3 (fT3) or free T4 (fT4) and are biologically active. 

Thyroid-stimulating hormone (TSH) is secreted by the pituitary gland and plays a major role in TH synthesis. It binds the TSH receptor (TSHR) on the thyrocyte basolateral membrane [39]. This activates a G-protein signal cascade which results in the intracellular rising of cyclic adenosine monophosphate. The ultimate consequence is the increase in TH synthesis. The TSH/TSHR link has many other effects. It up-regulates the NIS activity, thus raising the iodine trapping and stimulating the iodination of TG in the follicular lumen and the conjugation of tyrosine residues. It also increases endocytosis of the iodinated thyroglobulin protein across the apical membrane back into the follicular cell, proteolysis of iodinated thyroglobulin to form free T3 and T4, and secretion of T3 and T4 across the thyrocytes basolateral membrane. 

Three transcription factors are of the upmost importance for NIS gene transcription in the thyrocytes: NkX2.1, PAX8 and FOXE1. Nkx2.1 binds a proximal NIS promoter, up-regulating NIS activity [40,41]. PAX8 has the highest impact on NIS transcription, binding a distant region of the proximal promoter called the NIS Upstream Enhancer (NUE) [42,43,44]. Each promoter has one PAX8 binding site. In rodents, Nkx2.1 and FOXE1 are also able to bind the NUE, but this has not been proven yet in man [42,45]. Many other factors are able to enhance the NUE, such as a cAMP response element *(CRE-like)*, the GLI-similar 3 (GLIS3), the Nuclear Factor-kappa B (NF-kB), the Upstream Transcription Factor 1 (USF1). GLIS3 is essential in order to enhance the NIS transcription mediated by TSH, and the NF-KB p65 subunit seems to interact with PAX8 to increase NIS transcription [46,47,48].

## 3. Physiologic Metabolism during Pregnancy

Thyroid and iodine metabolism are modified during pregnancy to ensure proper maternal and fetal thyroid function. 

The human placenta is hemochorial with villous trophoblast; maternal and fetal circulations are fully separated by the placental barrier. Exchanges between these two circulations take place in the syncytiotrophoblast (SCT), where SCT cells are in contact with maternal circulation on their apical side and in contact with fetal capillaries on their basal side [49]. Different patterns are involved for substances to cross the placenta: passive transport (passive diffusion and facilitated diffusion), active transport with transporters, and endocytosis/exocytosis. Passive diffusion is the main placental transport mean for non-ionized liposoluble with low molecular weight (<500 Da) molecules. However, most of the molecules implied in iodine and thyroid metabolism require different means of transport because of their specificities. Placenta is permeable to iodine, anti-thyroid drugs (ATD), TSH Receptor Autoantibodies (TRAb), and Thyroid Peroxidase Antibodies (TPOAb). 

### 3.1. Iodine

Iodine requirement is rapidly increased by 50% during pregnancy in order to maintain maternal and fetal homeostasis [50]. As the fetus is unable to produce its own TH during the first trimester, it initially fully depends on maternal TH import. Maternal T4 production is thus rapidly increased by approximately 50% to meet both mother and fetal needs, which implies higher iodine needs. The fetal thyroid becomes fully functional after 17–19 WG but still requires adequate fetal iodine supply in order to operate [4,5]. Finally, median UIC is increased during pregnancy because of a physiological increased renal clearance. Different studies revealed that the median UIC was higher in pregnant women when compared to non-pregnant ones (for instance, 172 µg/L versus 132 µg/L for Stilwell et al.) [51,52,53,54]. Moreover, the percentage of pregnant women with UIC < 50 µg/L is inferior to the percentage of non-pregnant women (7.3% versus 16.8% for Stilwell et al.). The median UIC is believed to be constant throughout pregnancy, but this still has to be proven. 

The increased needs and the frequent iodine deficiency have led many scientific societies to recommend a systematic iodine supplementation with 150–250 µg of iodine/day for women who are pregnant, planning pregnancy, or breastfeeding. [55,56,57]. A median IUC of 150–249 µg/L is believed to indicate adequate iodine intake in pregnant women [58,59]. Unfortunately, ID during pregnancy persists and can cause adverse maternal and fetal outcomes, as will be discussed further. 

Maternal iodine crosses the placenta from the maternal circulation into the fetal circulation. NIS and Pendrin are deeply involved in this transport. Even though they were initially described in the thyroid, there is evidence of their presence in placental tissue [60,61,62]. The precise mechanisms involved in fetal iodine import remain unknown. Immunohistochemical analysis of the primary culture of villous cytotrophoblastic cells and of villous syncytiotrophoblast showed that Pendrin and NIS are mainly expressed respectively at the brush border membrane of SCT—facing the maternal blood—and in the cytotrophoblast (CT) [63]. However, these locations would imply an inverted iodine transport direction when compared to thyrocytes. On the contrary, a study on BeWo choriocarcinoma cells used as a model of iodine transport by placenta suggests more classic mechanisms, iodine being imported into the trophoblast through NIS and being exported into the fetal circulation through Pendrin [64]. 

NIS and Pendrin placental mRNA expression obtained using RT-PCR vary throughout pregnancy [63]. NIS mRNA expression is similar during the first and the third trimesters, whereas Pendrin mRNA expression is higher during the third trimester. This is consistent with the increased fetal iodine needs at term. Pendrin’s utmost importance is highlighted by Pendred syndrome. This autosomal recessive disease is caused by SLC26A4 mutation on chromosome 7q31 and makes Pendrin unfunctional [63]. It is characterized by severe hypothyroidism, goiter and variable deafness. 

Regulation of placental and thyroidal iodine transport is HCG-dependent. HCG (human chorionic gonadotrophin) is a specific to humans 37 kDa glycoprotein composed of two glycosylated subunits, non-covalently linked [65]. Alpha sub-unit contains 92 amino acids and is common with the alpha sub-units of other pituitary gonadotropin hormones (LH, FSH, and TSH) [66]. It contains two N-linked oligosaccharide sidechains. Conversely, the beta sub-unit is hormone specific. It is composed of 145 amino acid residues with two N-linked and four O-linked oligosaccharides [66]. Even though the HCG beta sub-unit confers the hormone’s biological specificity, it shares an overall three-dimensional structure with the TSH beta sub-unit. Indeed, both possess 12 half-cysteine residues, one N-linked oligosaccharide and three disulfide bonds at highly conservated positions [67]. In addition, glycoproteins hormone receptors of HCG and TSH are highly homogenous. They have a single polypeptide chain forming seven hydrophobic alpha-helices that constitute transmembrane segments [68]. 

The similarities between the beta sub-units and the receptors explain why HCG is able to cross-react with the TSH receptor [69]. In Jar and BeWo cells exposed to HCG, NIS expression and iodine import are increased [70,71]. On the contrary, they are decreased when HCG is withdrawn. 

Many different pregnancy-related hormones can affect iodine placental import. When exposed in vitro to HCG, oxytocin, and prolactin, primary cultures of term placental trophoblast cells increased Iodine 125 (I125) uptake by 60, 45, and 32%, respectively [72]. Accordingly to this increased I125 uptake, NIS mRNA was higher in exposed cells when compared to controls. Surprisingly, no increased expression of Pendrin was observed, which leads us to believe that other unknown mechanisms must be involved. 

It also appears that iodine uptake into the placenta is self-regulated by intracellular iodine. Indeed, placental NIS mRNA is upregulated in iodine-deficient rodents [73]. Conversely, NIS mRNA and iodine uptake are decreased when BeWo cells are previously exposed to iodine [71]. 

In order to provide the fetus with constant adequate iodine intake—in the event of inadequacies in maternal dietary intake—the placenta seems to be able to both store and transport iodine [74,75].

### 3.2. Maternal and Fetal Thyroid

#### 3.2.1. Maternal Thyroid Metabolism

Maternal thyroid activity is highly impacted by pregnancy for various reasons. As described earlier, HCG has a TSH-like effect on thyroid tissue [69]. This leads to a rise in fT4 synthesis and, conversely, to a decrease in TSH hypophyseal secretion by a negative feed-back [76]. The fT4 synthesis rise is temporary, lasting mostly during the first trimester. It ensures adequate fT4 fetal supply until the fetal thyroid is fully functional. Indeed, the fT4 synthesis diminishes when the HCG serum concentration falls at the end of the first trimester [76,77]. TSH is usually normalized in 2 to 3 weeks after the end of the negative feed-back phenomenon, but fT4 concentration constantly declines throughout the pregnancy [78,79]. 

Also, plasma volume expansion in pregnant women tends to increase the T3 and T4 pool size. In iodine-deficient areas, the physiological rise of iodine renal clearance can be responsible for a decrease in TH synthesis. 

Finally, TBG hepatic synthesis is increased because of the excess estrogen during pregnancy [77,80]. An increase of sialic acid content in the carbohydrate chains during its synthesis also prolongs its half-life. This results in a rise in T3 and T4 maternal serum concentrations. 

#### 3.2.2. Fetal Thyroid

The fetal thyroid gland develops from a thickening of the pharyngeal floor from the median oral endoderm and from two caudal extensions of the fourth branchial pouches at approximately day 24 of human gestation [81]. The thyroid is initially located at the foramen caecum, which is the junction of the anterior two-thirds and posterior one-third of the tongue. At four WG, the embryonic structures are fused, and the thyroid gland consists of two lateral lobes with superior and inferior poles connected by a median isthmus [82]. When the isthmus is absent, the two lobes are distinct from each other. Between five and seven WG, the thyroid migrates to its almost definitive position between the second and the fourth cervical vertebrae [83]. This descent through the neck tissues mainly happens during the first trimester and follows the thyroglossal duct. In 50% of the cases, the distal part of the thyroglossal duct differentiates into the additional pyramidal lobe of the thyroid gland [82]. The rest of the duct is afterward obliterated before the end of the first trimester in most cases. After the first trimester, the thyroid migration drastically slows down but still continues throughout the pregnancy. Its final site between the 5th cervical and the 1st thoracic vertebrae is probably reached only in adults [83].

Alongside its migration, thyroid tissues are functionally differentiated [84,85]. The first stage of this differentiation takes place between seven to nine WG. At that time, the thyroid is only composed of unpolarized thyrocyte precursors developed from the medial endoderm, which are unable to synthesize TH. Between 10 and 11 WG, small follicles are formed by polarizing thyrocytes, which allow the fetal thyroid to accumulate iodine and synthesize TH. In parallel, calcitonin-secreting cells (also known as parafollicular or C cells) are developed from the fourth branchial pouches [40,86]. By 12 WG, the thyroid organogenesis is complete. 

The NIS gene and the NIS gene transcription factors (nkx2.1, FOXE1 and PAX8) are highly involved in fetal thyroid development [85]. Trueba et al. studied human embryos and fetuses from seven to 33 WG with quantitative PCR to analyze mRNA expression [87]. They proved that FOXE1, PAX8 and NKX2.1 were constantly expressed from seven to 33 WG. NIS mRNA was the last to be detected at eight WG, but their quantity greatly rose between nine and 10 WG, underlining NIS’s key role in fetal thyroid function. 

Fetal TH synthesis begins around 10 WG, and fetal T4 can be detected in the fetal circulation by 11 WG [5,6]. It is fully dependent on the maternal iodine transport through the placenta. However, it is not entirely functional before 17–19 WG [5]. Until then, maternal TH are mandatory for fetal physiological development, and the fetus relies entirely on them in the first trimester [4]. 

After the beginning of the second trimester, TH from both maternal and fetal thyroid glands is present in the fetal circulation. The fetal concentration of total and free circulating T3, T4 and TGB increase throughout pregnancy and reach the mean adult values at approximately 36 WG. This reflects the increased maturation of the fetal pituitary gland and liver [6]. However, fetal TSH is also increased even when TH are high, suggesting that the fetal pituitary gland sensitivity to negative feed-back is limited.

### 3.3. Thyroid Metabolites Placental Transport

#### 3.3.1. TH Placental Transport

Placenta is the interface between the mother and fetal circulations. It is poorly permeable to T4 and T3, into and out of the fetal circulation [5]. However, fetuses with athyreosis display at least one-third to half of the mother’s fT4 serum concentration. This indicates a potential consequent mother-to-fetus TH passage under high gradient concentration. Transplacental T4 circulation seems to be asymmetrical, according to an in vitro study [88]. The maternal-to-fetal passage is limited, but the fetal-to-maternal is way faster. Placenta could thus play a protective role in order to avoid too high T4 concentration in fetal circulation, but more studies are needed. 

The exact mechanisms of TH import across the placenta and into the fetal circulation still have to be studied. TH bound to TGB, transthyretin, or serum albumin in the maternal serum are transported to the placenta [89]. Afterward, cellular membrane transporters are in charge of carrying TH into and across the placenta.

Placental transport patterns vary throughout the pregnancy. From five to six WG to the end of the first trimester, the exocoelomic cavity is a privileged area for maternal-fetal exchange. TH are carried across the placenta into the exocoelomic cavity and then pass into the fetal circulation through the yolk sac and then the fetal intestines [90,91]. The yolk sac is an extension of the fetal intestinal circulation and is able to absorb and deliver different proteins—such as TH—to the fetus. Early T3 and T4 concentrations in fetal tissues are more than 100-fold times lower than in the maternal serum [5]. However, the concentrations of fT4 in the coelomic and amniotic fluid are equivalent to approximately a third of the maternal one, reaching values high enough to induce biological effects.

From the second trimester, the placenta becomes the main area of materno-fetal exchange. TH are transported directly into the fetal blood via the placenta after crossing cytotrophoblast and/or syncytiotrophoblast [5].

TH transporters are involved in order for TH to cross the placenta. Their presence in human choriocarcinoma cell lines has been known for over 30 years [92]. More than 15 TH transporters have been identified, but only a fraction of those are involved in placental traffic [93,94]. mRNA sequencing of human placentas showed that L-type Amino-acid Transporters (LAT1 and LAT2), Mono-Carboxylate Transporters (MCT8 and MCT10), and Organic-Anion Transporting Peptides (OATP1A2 and OATP4A1) are the ones playing an active role in transporting TH to the fetus [95]. They are located on the plasma membranes of cytotrophoblasts and syncytiotrophoblast, and each one of them can interact with TH. Studies are still ongoing in order to determine how they link and transport TH. For instance, the T3 carboxyl group could be bound by H-bonds to a positively charged arginine and a histidine at the substrate-binding center of MCT8, but the exact transport mechanism remains unclear [96,97].

Table 1 describes TH transporters’ locations in the placenta and their variations throughout pregnancy [94,98,99,100,101,102,103,104,105,106,107,108,109].

#### 3.3.2. TSH

Placenta is usually considered to be impermeable to TSH. Cord blood TSH measurements in infants born with congenital absence of the anterior pituitary gland show very low TSH levels [110]. This suggests that maternal TSH cannot cross the placenta. However, an in vitro study of dually perfused human term placenta proved that TSH actually crosses both the placenta and the fetal membranes, but so sparingly that it cannot influence fetal thyroid metabolism [111]. TSH is present in amniotic fluid from 18 WG, and its fetal origin is confirmed by the high correlation between amniotic fluid and blood cord concentration [112]. Fetal TSH level slowly rises to a peak value of 15 mU/L at 25 WG [113]. Whether it remains stable or slightly decreases during the last trimester remains debated [114]. 

#### 3.3.3. TRH

TRH is a hypothalamic-releasing factor produced by paraventricular neurons in the hypothalamus. After being produced and activated in the anterior pituitary, it binds TRH-receptors, inducing the synthesis and secretion of the TSH beta-subunit by the hypophysis. 

Placenta is highly permeable to maternal TRH. It is present in the fetal brain way before the fetal hypothalamus is developed [115]. The fetal hypothalamus and hypophysis are functional from 20 WG, but TRH can be detected in fetal blood as soon as 12 WG. Maternal TRH could influence fetal TSH synthesis, but this is uncertain. TRH deprivation in rodent studies is not responsible for altered TSH synthesis, but it seems to affect fetal TSH after 37 WG [114,116].

Interestingly, TRH accelerates fetal lung maturation in rodents and increases the amount of surfactant secreted using extra-thyroidal pathway stimulation [117]. However, the widespread use of antenatal corticosteroids has supplanted that of TRH in this indication. Interestingly, TRH accelerates fetal lung maturation in rodents and increases the amount of surfactant secreted using extra-thyroidal pathway stimulation [117]. However, the widespread use of antenatal corticosteroids has supplanted that of TRH in this indication. 

#### 3.3.4. TRAb/TPOAb

Thyroid antibodies (TRAb and TPOAb) are both class G immunoglobulins (Ig G). They have a tetrameric T-like shaped structure containing two heavy and two light chains. Each IgG weight of approximately 150 kDa consists of an antigen-binding site (Fab) and a constant region (Fc) [118]. Fc binds Fc receptors on a wide variety of specific targets. 

Studies revealed that Fab and Fc do not cross the placenta in a similar way. When radiomarked and injected into pregnant women, Fc and complete IgG transport are greatly more efficient than Fab transport alone [119]. This lead to the discovery of placental FcRN [120,121]. FcRN is a membranal protein of 40–45 kDa, non-covalently linked to a b2-microglobulin. It is highly sensitive to pH as its ligand affinity is a hundred times higher when exposed to neutral than acid pH (7.4 versus 6).

Placental FcRn is poorly expressed in CT. It is preferentially located in SCT and in the fetal capillary endothelial cells [122,123]. IgG placental transfer requires both FcRn and endocytosis [124,125,126]. FcRn binds IgG on the placental maternal side, and the FcRn-IgG complex is imported into and carried across SCT by endocytosis. The acid environment in the vesicle reinforces the FcRn-IgG link and protects it from lysosomal degradation. At the SCT basal side, the FcRn-IgG complex is freed from the vesicle and dissociated by the physiological neutral pH. IgG is transferred across the SCT basal membrane directly into the fetal capillaries of the placental villi and indirectly after stromal transit. 

IgG transplacental transport begins at approximately 13 WG. Fetal IgG serum concentration increases throughout the pregnancy, starting at 5–10% of maternal blood concentration at 17–22 WG, reaching 50% of maternal blood concentration by 32 WG and finally exceeding maternal blood concentration (120–130%) at birth [127,128]. 

TRAb and TPOAb require active transport in order to cross the placenta because of their high molecular weight. This placental transport is mandatory for fetal metabolism and implies the FnCR [129,130]. However, the specific thyroid antibody pathways involved in placental import have not been identified yet.

### 3.4. Deiodinases

Deionidase are a family of selenoproteins. Each one of them has specific tissue regulation and distribution, and both deionidase 2 (D-II) and 3 (D-III) have been identified in the human placenta [131]. D-II is located in CT cells and inconstantly in SCT during the first trimester. D-II binds preferably T4 over rT3 and converts it into active T3 [132]. D-III is conversely located in SCT and is able to catalyze inner ring deiodination of T3 and T4, inactivating T3 to T2 or T4 to rT3 [133]. It has a higher affinity for T3 than D-I.

Placental D-III is much more active and highly expressed than D-II (approximately 200 times at the beginning of the pregnancy and up to 400 times at term). Both their activity and expression decrease throughout pregnancy and seem to be regulated by fetal circulating T4 [134,135]. This could explain why fetal T3 levels are relatively low and fetal rT3 are high. Polak suggested that this mechanism could cause low in-utero thermogenesis [114]. 

Deionidase is also expressed in fetal tissues. For instance, D-II is expressed in the fetal brain. It is believed to protect the fetal brain from hypothyroidism [136]. 

## 4. Maternal Pathologies during Pregnancy

### 4.1. Gestational Transient Hyperthyroidism (GTH)

HCG cross-stimulation of the TSH receptor is responsible for gestational transient hyperthyroidism in 2.4–11% of pregnant women [137,138]. It is usually more common between eight to 11 WG than 12–14 WG, as the HCG decreases at the end of the first trimester. The GTH is, by definition, transient, resolving with the decline of HCG. TSH concentrations are normalized in less than 3 weeks [138]. The more severe conditions are caused by hydatidiform moles/choriocarcinomas or multiple pregnancies because a large amount of HCG is secreted in those cases [139]. 

GTH is often revealed by pregnancy symptoms being more intense and severe than usual. In addition to asthenia and nausea, it may provoke tremors, tachycardia, vomiting, and weight loss (up-to hyperemesis gravidarum). 

GTH is seldom responsible for thyrotoxicosis. In those rare cases, ATD might be necessary in order to treat the symptoms and to rapidly lower the maternal TH blood concentration [140]. If the ATD do not properly control the hyperadrenergic symptoms, beta-blockers can be used in addition [141]. These medications should be used with caution because they do cross the placental and can be responsible for neonatal hypoglycemia and/or bradycardia [142].

### 4.2. Gestational Transient Hyperthyroidism (GTH)

Graves’ disease is an auto-immune disease caused by a certain type of TSH Receptor Antibodies (TRAb), which binds and stimulates TSHR [143]. Inhibiting TRAb also exist but only represent less than 10% of the total TRAb and do not play any role in Graves’ disease. It affects approximately 0.2% of pregnant women and is rarely discovered during pregnancy [144]. Graves’ disease is often revealed by rather unspecific symptoms that are shared with GTH. The TRAb increase during the first trimester and a co-existing GTH can both worsen the symptoms at the beginning of the pregnancy [145]. Post-partum is also a period at risk for thyrotoxicosis because of potential immune system rebound [146].

Uncontrolled thyrotoxicosis in cases of Graves’ disease is associated with miscarriage, gestational hypertension and pre-eclampsia, thyroid storm and maternal congestive heart failure [147,148].

As for non-pregnant women, ATD are the main treatment for GH [141]. The objective is to maintain total T4 under 150% of the non-pregnant upper limit value and fT4 under the upper limit value. The doses should be lowered when T3 and T4 values are corrected and, ultimately, when TSH becomes detectable. A thyroidectomy is an option during the second trimester if a woman has an uncontrollable GH, a highly symptomatic goiter, or cannot tolerate ATD [148].

### 4.3. Distinguishing GHT from Graves’ Disease

It is crucial to be able to distinguish Graves’ disease from GTH. Women affected by Graves’ disease usually present familial and personal history of auto-immune diseases. The presence of three other elements is highly in favor of Graves’ disease: maternal goiter, ophthalmopathy and TRAb presence. In addition, some maternal biological and sonographic markers may be helpful [149]. Usually, in Graves’ disease, the fT3/fT4 ratio is >2.7, the total T3/total T4 ratio is >20, TSH is <0.01, and HCG are <70,000 mUI/mL [149,150]. fT3/fT4 ratio is the best biological parameter. Thyroid ultrasound can measure the mean Systolic Peak Velocity of the Superior Thyroid Artery (STA-PSV) and the Superior Thyroid Artery Diastolic internal diameter (STA-D). STA-PSV < 40 cm/s and STA-D > 2.0 mm are associated with Graves’ disease, whereas STA-PSV > 40 cm/s and STA-D are associated with gestational transient hyperthyroidism [151,152]. 

### 4.4. Thyroid Storm

Thyroid storm is a scarce condition that affects less than 1% of pregnant women with hyperthyroidism [147,153]. It can be caused by an inciting event such as surgery, infection, pre-eclampsia, or labor. Atrial fibrillation, congestive heart failure and liver failure can develop, with mortality rates up to 25% [154]. It is therefore recommended to preventively treat all hyperthyroid pregnant women, especially before undergoing surgery [155]. 

### 4.5. Maternal Hypothyroidism

The prevalence of maternal hypothyroidism during pregnancy ranges between 1–2% [130]. Worldwide, the most common cause of maternal hypothyroidism is ID. Indeed, the thyroid usually uses its iodine stores to compensate for any insufficient supply. An iodine-deficient woman becoming pregnant can easily experience hypothyroidism as their iodine needs suddenly and rapidly increase. In areas of iodine insufficiency, the main etiology is autoimmune disease (especially Hashimoto’s thyroiditis caused by TPOAb) [156]. The other causes of hypothyroidism during pregnancy are thyroidectomy, ablative iodine therapies and over-treatment of a GTH [157].

Maternal hypothyroidism can lead to adverse pregnancy outcomes, such as miscarriage and gestational hypertensive disorders, including preeclampsia, abruption placenta and post-partum hemorrhage [158,159,160,161]. The risk of adverse outcomes in chronic autoimmune thyroiditis is higher than in ID hypothyroidism, and TPOAb-positive mothers have an increased risk of placental malperfusion irrespective of their TSH serum concentrations [162]. Isolated maternal hypothyroxinemia (serum low free thyroxine and normal thyroid stimulating hormone levels) is also associated with maternal and neonatal adverse outcomes such as premature rupture of membranes and low Apgar scores at birth [163].

## 5. Fetal Consequences of Pathological Thyroid Metabolism

### 5.1. Fetal Anomalies Caused by Hyper or Hypothyroidism

TH perfect balance in the fetal compartment is essential to ensure normal embryogenesis, brain development and fetal growth. 

#### 5.1.1. Congenital Heart Defects

Rodents with fetal hyperthyroidism induced by D-III knock-out are more likely to present congenital heart defects than those with normal thyroid metabolism. Three hundred and 64 misregulated genes were identified in D-III knock-out hearts with RNA sequencing, such as TBX5, MEF2C, TGFB1, TP53, and FOXO4 [164]. Dong et al. confirmed the relationship between hyperthyroidism and congenital heart defect in humans [165]. They proved that high concentrations of fT4 measured between 12 and 18 WG are associated with an increased risk of congenital heart defects. In their work, each 1% of free-to-total thyroxine proportion was associated with a 1.41-fold higher risk of congenital heart defect. 

#### 5.1.2. Impairments to Brain Development

Neurological impairment is a negative outcome of fetal hypothyroidism. Neuronal proliferation, neuronal migration, axonal growth, dendritic synaptogenesis, neural differentiation and migration and myelination require adequate levels of TH [4]. For instance, neural differentiation of neural stem cells depends on sequential molecular cascade [166]. Pax6, a sequence-specific DNA binding transcription factor, binds the Tbr2 enhancer region and induces its expression [167]. Tb2r is a T-box transcription factor, and neural stem cells differentiate into intermediate progenitor cells under its influence [168]. Tbr2 up-regulates Tbr1 expression, which induces intermediate progenitor cells’ differentiation into postmitotic projection neurons. This molecular cascade could be even more complex, implying Neurog2, Insm1 and Neurod1. Maternal TH deficiency in rodents down-regulates Pax6, thus diminishing Tbr2 expression. This results in the persistent decrease of intermediate progenitor cells and reduces neuron-specific nuclear protein positivity in the first and third layers of the neocortex, which reflects a depletion in mature neurons. The main consequence of this pathological phenomenon is reduced cortical thickness, which indicates a non-compensatory impairment in neurogenesis (Mohan 2012) [169]. Congenital hypothyroidism in mice highly also alters the subventricular zone—which is the largest neural stem cell harbor area in the adult mammalian brain—and is responsible for underperformances on short-term memory tests [170]. Finally, Shimokawa and al. proved that congenitally hypothyroid rodents show retardation of cerebellar morphogenesis (poor dendritic arborization of Purkinje cells and retarded migration of granule cells) [171]. These are responsible for severe impairment of motor coordination and balance. They also proved that congenitally hypothyroid rodents show intense anxiety and depression, which could be explained by deterioration in the axonal transport of dopamine in the nigrostriatal pathway.

As the fetus is fully dependent on maternal TH during the first trimester, pathological variations of the maternal serum TH concentration can also result in neurodevelopmental disorders. For instance, high maternal T3 and both high or low maternal fT4 are associated with an increased risk of weakened neuropsychological development and attention-deficit hyperactivity disorder in infancy [172,173].

#### 5.1.3. Birthweight

In addition to their role in heart and brain development, TH are of the utmost importance in birth weight regulation. Indeed, maternal subclinical hypothyroidism during pregnancy is associated with a higher risk of low birth weight, and isolated hypothyroxinemia is associated with a higher risk of high birth weight [174]. The relationship between maternal TH serum concentration and birthweight could be mediated by triglycerides, as triglycerides are correlated with maternal fT4 and neonatal birthweight [175,176]. Maternal carnitine could also be involved in this process because its serum concentration during the second trimester–alongside fT4–has a negative influence on birthweight [177]. Precise mechanisms involved in this process still have to be elucidated.

### 5.2. Fetal Hyperthyroidism 

Congenital hyperthyroidism is less frequent than congenital hypothyroidism, but its consequences can also be disastrous. The most common cause is maternal Graves’ disease. As described earlier, TRAb cross the placenta and stimulate fetal TSHR, leading to an increase in fetal TH secretion. As the fetal TSHR is able to respond to TSH stimulation from 17–19WG, fetal hyperthyroidism develops during the second trimester, especially in women with high levels of TRAb [178]. It can lead to fetal and then post-natal thyrotoxicosis. The maternal TRAb can take as long as 4 months to disappear from the child’s blood circulation. 1% of children born of women with Graves’ disease present hyperthyroidism [179].

Two other causes of fetal hyperthyroidism are TSHR mutations and McCune-Albright syndrome. They both result from signal transduction modifications in the fetal thyroid. 

Familial nonautoimmune hyperthyroidism (FNAH) and Sporadic nonautoimmune hyperthyroidism (SNAH) are two types of activating TSHR germline mutations. The mutated TSHR, in a pathway involving GTP-binding proteins, adenylate cyclase, cAMP, cAMP-dependent protein kinase, phospholipase C, diacylglycerol and calcium, increases the TH synthesis and secretion with no feedback loop [180]. 

FNAH is an autosomal dominant disorder that has never led to a prenatal hyperthyroidism diagnosis. The first hyperthyroidism symptoms indeed appear from 1 to 23 years old [180,181]. 

SNAH is caused by de novo mutations. Seventeen patients have been reported in the literature with 12 identified mutations. Interestingly, two cases presented prenatal features (fetal tachycardia in one case and a goiter associated with craniosynostosis in the other one), which raised suspicions [182,183]. 

Among various other manifestations, McCune-Albright syndrome can cause hyperthyroidism. This syndrome results from activating mutations at the R201 position in the GNAS gene, which encodes the alpha subunit of G proteins [184]. Cases with neonatal hyperthyroidism have been reported–disturbing the same signaling pathway as the TSHR mutations described above–but fetal hyperthyroidism was not suspected [185,186,187]. However, McCune-Albright syndrome has been described prenatally in a fetus with ovarian cysts [188]. It should thus be evoked in fetal hyperthyroidism when there is no argument for maternal Graves’ disease. 

**Figure 1:**
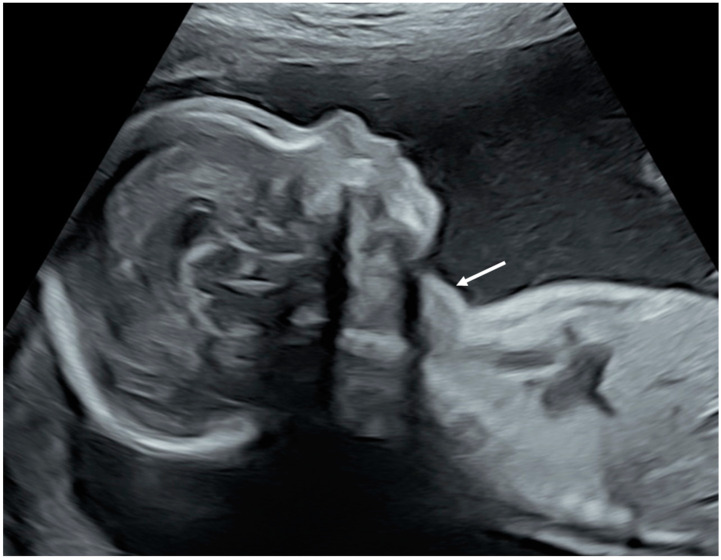
Sonographic view of fetal thyroid goiter from a sagittal plane (personal data from Bicetre Hospital Fetal Medicine Unit). The arrow points toward the fetal neck, which is distorted by a hyperthyroidic goiter.

Classic neonatal and fetal hyperthyroidism include fetal tachycardia and goiter. Hepatomegaly, splenomegaly, gynecomastia and cardiac insufficiency can also occur [189]. Fetal hyperthyroidism must be carefully looked for in fetuses from mothers with Graves’ disease, McCune-Albright and FNAH/SNAH. Prenatal sonographic features such as goiter with central vascularization, tachycardia, hepatomegaly, splenomegaly and cardiac insufficiency can easily be diagnosed (Figure 1) [190]. Fetal thyroid size curves are available in order to diagnose fetal goiter [191,192]. Fetal therapy for hyperthyroidism consists of administering ATD to the mother because they can cross the placenta [189].

### 5.3. Fetal Hypothyroidism

Congenital hypothyroidism affects one in 4000 newborns. The most frequent causes of neonatal hypothyroidism are thyroid dysgenesis, which consists of an impairment of the thyroidal gland development, and thyroid dyshormonogenesis, which consists of thyroid hormone synthesis disorder [193]. Dyshormonogenesis is usually caused by genetic disorders (mainly TPO mutations but also thyroglobulin mutations, NIS mutations, Pendred syndrome, Allan–Herndon–Dudley syndrome, etc.), ATD overload (especially when over-correcting GHT), iodine overload by iodine-based medicine consumption, maternal, or TSH blocking antibodies [194]. TPOAb can cross the placenta, but unlike TRAb, they cannot cause fetal dysthyroidism. 

**Figure 2:**
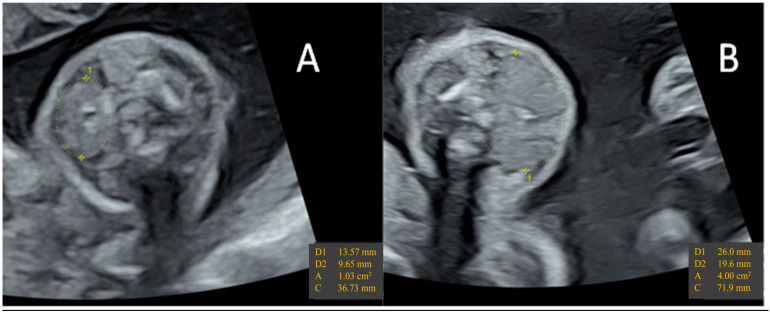
Sonographic views of fetal thyroids in an axial plane at 23 WG (personal data from Bicetre Hospital Fetal Medicine Unit). Image (**A**) represents a normal fetal thyroid with a transverse diameter of 13.57 mm and a perimeter of 36.73 mm, whereas image (**B**) represents a large hypothyroidic fetal goiter with a transverse diameter of 26.0 mm and a perimeter of 71.9 mm.

Hypothyroidism may manifest in the fetus only in the event of dyshormonogenesis or ATD overload. Fetal hypothyroidism is responsible for delayed bone age, surprisingly increased fetal movements and eventually bulky goiter with peripheral vascularization (Figure 2) [190,195]. Fetal blood sampling by cordocentesis (percutaneous umbilical blood sampling) is often needed to confirm hypothyroidism and dosing fetal TSH, T3, and T4 [189]. After the confirmation of fetal hyperthyroidism, prenatal treatment can be undertaken. It consists of repeated intra-amniotic injections of thyroxine.

Severe ID is the main cause of post-natal endemic “cretinism.” Two types of this condition have been described. The neurological one is characterized by intellectual disability with an intellectual quotient around 30, mutism, and spastic diplegia. The myxoedematous one is characterized by intellectual disability—however less severe than the neurological one—and delayed bone age [196]. Both are caused by hypothyroidism, which affects fetal neurological development.

Allan–Herndon–Dudley syndrome is another example of the utmost importance of maternal TH for fetal brain development and, thus, of placental TH transporters. It is a singular syndrome caused by mutations in the Solute Carrier Family 16, Member 2 (SLC12A2) gene on the X chromosome [197]. This gene encodes for the MCT8. The mutations include truncations, in-frame deletions, nonsense and missense mutations. They lead to central hypothyroidism and peripheric hyperthyroidism. Allan–Herndon–Dudley syndrome manifests in boys as congenital hypotonia, which is detectable in the early weeks/months of life. It also causes severe intellectual deficits and possibly a distinctive dysmorphism. fT3 serum concentration is abnormally high, whereas fT4 can be low or normal, and TSH is slightly elevated or normal. 

## 6. Conclusions

Iodine and thyroid role in pregnancy is central. Any impairment in their metabolism can affect both the mother and the fetus and eventually put at risk the newborn development. Iodine, thyroid hormone and thyroid metabolites in the fetal compartment are precisely regulated, and the placenta is highly involved in this regulation. 

This review underlines the utmost importance of iodine and thyroid metabolism during pregnancy and the need for careful screening and treatment of mothers, fetuses and new-born to ensure optimal health. 

## Figures and Tables

**Table 1:** TH transporters’ locations in the placenta and their variations throughout pregnancy [94,98,99,100,101,102,103,104,105,106,107,108,109].

	Placental Location	Evolution throughout Pregnancy			Substrates Uptake	Substrates Efflux
		T_1_	T_2_	T_3_		
LAT1	SCT apical surface	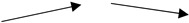			T2 > rT3 > T3 > T4	T2
LAT2	SCT	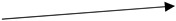			T2 > T3	*
MCT8	CT + SCT	*			T3, T4 > rT3 > T2	T3, T4
MCT10	CT + SCT at T_1_ SCT at T_3_	*			T3 > T4	T3
OATP1A2	SCT basal surface CT and CT EV	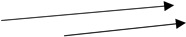			T3 > rT3, T4	*
OAPT4A1	SCT apical surface	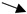			T3 > T4, rT3	*

T2: Diiodothyronine; T3: Triiodothyronine; rT3: Reverse Triiodothyronine; T4: Thyroxine; LAT1: L-type Amino-acid Transporter 1; LAT2: L-type Amino-acid Transporter 2; MCT8: Mono-Carboxylate Transporters 8; MCT10: Mono-Carboxylate Transporters 8; OATP1A2: Organic-Anion Transporting Peptide 1A2; OATP4A1: Organic-Anion Transporting Peptide 4A1; CT: Cytotrophoblast; EV CT: Extra-villous Cytotrophoblast; SCT: Syncytiotrophoblast; T_1_: first trimester of pregnancy; T_2_: second trimester of pregnancy; T_3_: third trimester of pregnancy; *: no data available.
